# Performance of Different Urban Design Parameters in Improving Outdoor Thermal Comfort and Health in a Pedestrianized Zone

**DOI:** 10.3390/ijerph17072258

**Published:** 2020-03-27

**Authors:** Xuan Ma, Mengying Wang, Jingyuan Zhao, Lei Zhang, Wanrong Liu

**Affiliations:** 1 Department of Architecture, Chang’an University, Xi’an 710061, China; mxozil@yahoo.com (X.M.); zl.wc@chd.edu.cn (L.Z.); Loislwr1995@163.com (W.L.); 2 Graduate school of Human-Environment Studies, Kyushu University, Fukuoka 8190379, Japan

**Keywords:** Urban design parameter, Pedestrianized Zone, Thermal comfort, Measurement survey, Numerical simulation

## Abstract

Global climate change and urban heat islands have generated heat stress in summer, which does harm to people’s health. The outdoor public commercial pedestrianized zone has an important role in people’s daily lives, and the utilization of this space is evaluated by their outdoor thermal comfort and health. Using microclimatic monitoring and numerical simulation in a commercial pedestrianized zone in Tai Zhou, China, this study investigates people’s outdoor thermal comfort in extreme summer heat. The final results provide a comprehensive system for assessing how to improve outdoor human thermal health. Under the guidance of this system, local managers can select the most effective strategy to improve the outdoor thermal environment.

## 1. Introduction

The rapid development of urbanization in China brings not only convenient lifestyles, but also the serious deterioration of living environments, such as the urban heat island in the summer, which can negatively influence human thermal comfort [1]. The term ‘thermal comfort’ refers to “the conditions of the inner mind that express satisfaction with the thermal environment”. An extreme outdoor environment will adversely influence outdoor public health, especially that of the elderly who are more sensitive to heat stress [2]. Human thermal comfort is largely determined by different meteorological parameters, including wind velocity, air temperature, relative humidity and mean radiant temperature [3]. All of these together can alter the energy exchange of the human body through radiation, conduction and convection.

The commercial pedestrianized zone can provide citizens and tourists with entertainment and socialization, and the environmental conditions in this region can improve people’s recreational and living activities, so the quality of the thermal environment needs to be discussed [4]. In accordance with previous studies, people’s thermal comfort in the outdoor environment will be affected by different urban design parameters including aspect ratio (H/W, H is the average height of the building and the W is the width of the street), sky view factor (SVF), street orientation, urban vegetation and paving material of ground surface. The aspect ratio (H/W) expresses the ratio between the average height of the building and the street width [4], several studies [5,6,7] have shown that increasing building average height (aspect ratio), impeding solar radiation and providing shading can contribute to ameliorate the thermal environment. The second factor is the sky view factor (SVF), which is expressed as “the ratio of the sky which can be seen from a stable position on a surface to that potentially available” [8], this index is a number ranging from 0 to 1. A previous study has shown that a lower SVF brings lower daytime temperatures in canyon space [9]. In addition, SVF also affects the level of the wind speed, and a study found that a 10% increase in SVF will lead to an 8% increase in wind speed [10]. The street orientation is considered as the third factor for influencing the thermal environment, it defines the standard of solar access to the inner street, a previous study found that the mutual shading on the west and the east is the main reason for a lower air temperature in a north–south-oriented street in the afternoon [11]. As well as the aforementioned three factors, the urban vegetation is another significant factor for improving people’s thermal sensation in summer, which cools down the environment through shading and evapotranspiration. A study conducted in Hong Kong shows that a 25%–40% increase in the percentage of trees will reduce the daytime heat island by 0.5 °C [12], a 20% increase in the number of trees in the campus of Saga University, Japan, decreases the average maximum temperature by 2.27 °C in the summer [13] and field measurement in the hot-humid climate zone of Singapore shows a difference of 2.0 °C between tree canopy and ambient area [14]. Also, changing the paving material of the ground surface with higher albedo is another strategy to alleviate heat stress. A study in the hot-humid climate zone of southern China shows that the 2% increase in paving material with higher albedo will reduce by 0.3 °C in the outdoor environment [15].

While previous studies have discussed the cooling effects of different urban design parameters, most of them were evaluated separately, and a comprehensive system to evaluate the relative importance of different parameters in the urban built environment is lacking, especially in the commercial pedestrianized zone. In this study, the field survey and numerical simulation are conducted to assess the cooling effect of each different parameters, and the final findings will put forward a comprehensive standard for helping the local managers and policy makers to choose the best strategy to improve outdoor thermal comfort and health.

## 2. Methodology

### 2.1. The Methodological Framework

**Figure 1:**
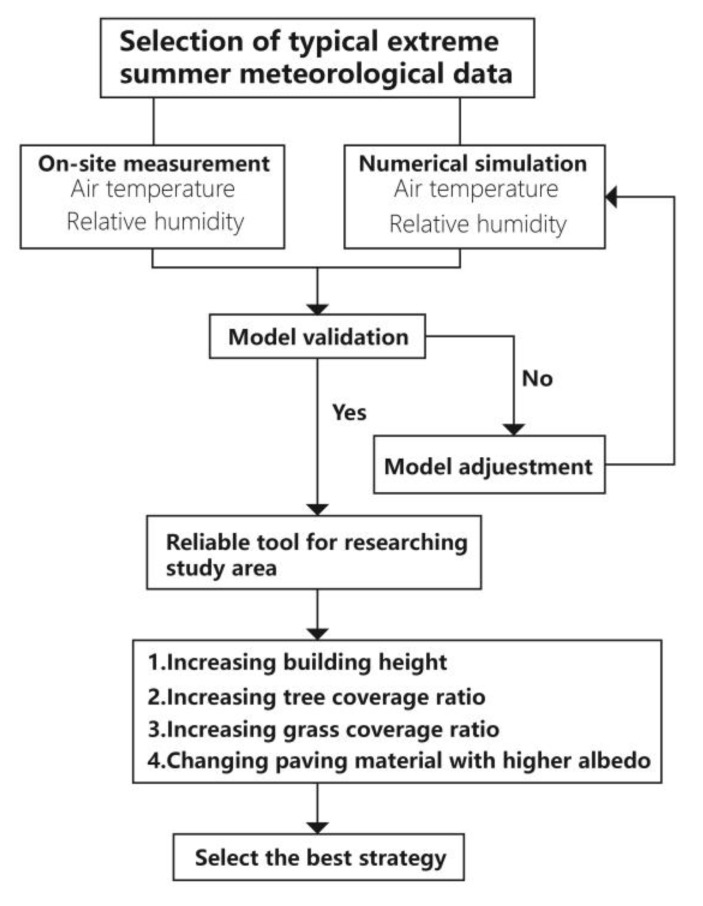
Methodological framework of the current study.

The methodological framework of this study is shown in Figure 1. On-site measurement is carried out, where the measured data are compared to the output results of ENVI-met by linear regression and the index RMSE (root mean square error) to validate the simulated performance. After that, we put forward some scientific hypotheses to understand the effect of different mentioned urban design parameters in cooling the thermal environment, this helps to the select the nest strategy to improve people’s thermal comfort and health.

### 2.2. Research Area and Field Survey

**Figure 2:**
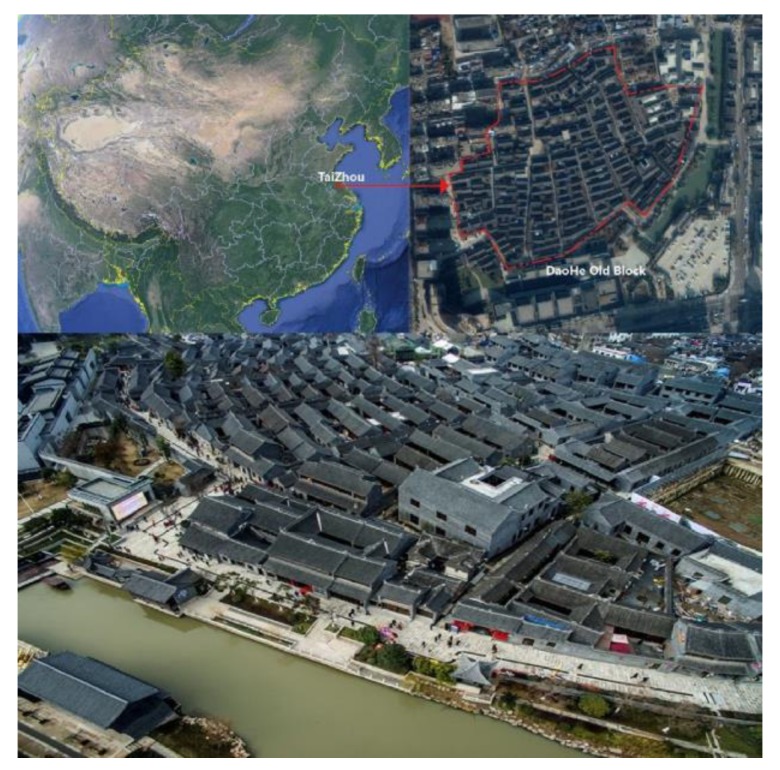
The location of the research site.

Due to the effect of the monsoon climate, Tai Zhou city (Southern China) is very hot and humid in the summer [16]. The current study was conducted in Dao He Old Block, which is a Chinese traditional architectural settlement from ancient times and is now a famous scenic spot of the city that attracts many tourists every year [17] (Figure 2).

The on-site measurements of the field survey were carried out on 30 and 31 July 2016 between 9:00 am and 5:00 pm. According to the published meteorological information of the local administration, the hottest time of a year appears in July and the maximum can reach 37 °C [16]. The data of air temperature and relative humidity were collected by a stable microclimate machine (TR-72wf), its accuracy was 0.1 °C for air temperature and 0.1% for relative humidity. In addition, the wind velocity was recorded using an anemoscope, which also had high accuracy. Table 1 shows the detailed meteorological data of the two measured days.

**Figure 3:**
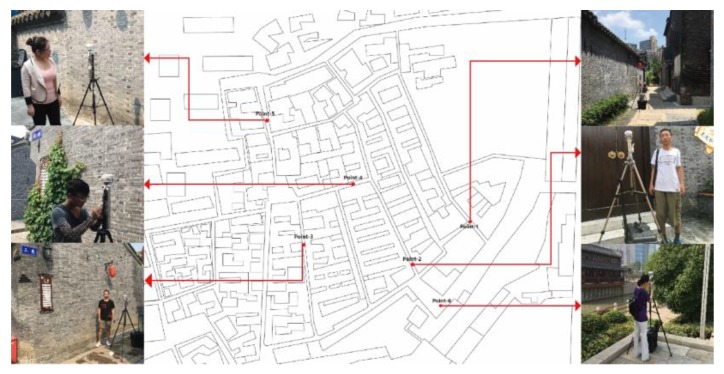
The selected points of this study [18].

Considering the historical meaning and the importance of the heritage, the whole zone is divided into six parts for collecting data in accordance with different geometry, each point has typical meaning in this study [18] (Figure 3).

**Figure 4:**
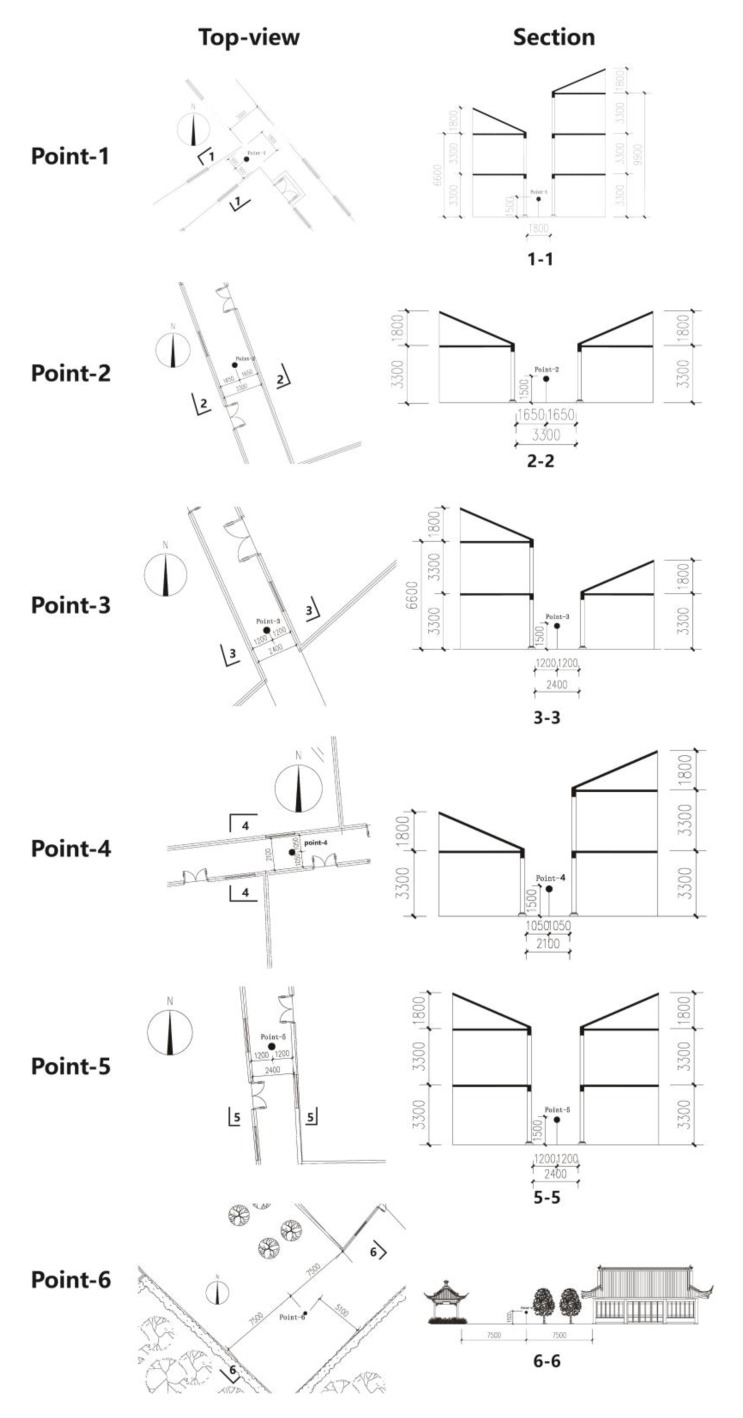
The plane and section of selected points [18].

The selected first point (point-1) is in a northwestern street, aspect ratio (H/W) being 4.6, and this point has the highest aspect ratio in this commercial zone. Like point-1, point-2 is also a northwestern street with a different H/W, being 1. Different from the former two points, the third point (point-3) is in a north–south directional street, with the H/W being 2.3. Like point-3, point-5 is also located in a north–south directional street with a higher H/W, being 2.75. In addition, point-4 is in the unique north-western street of the research site, and the last point (point-6) is located in an open space, which is covered by a little vegetation (Figure 4 and Table 2). 

**Figure 5:**
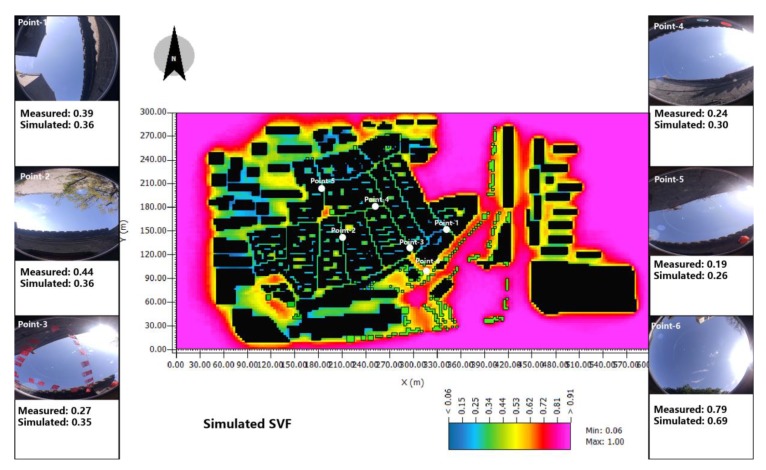
The validation between measured and simulated sky view factor (SVF) [18].

Another step to ensure the accurate geometry of the selected points in the model built by ENVI-met is a comparison of the simulated and measured SVF. The results of the measured results of the SVF are calculated by the software Ray-man, which can calculate the SVF through the hemisphere photo captured by a fish-eye camera. The simulated result can be conducted by the ENVI-met. The final validation between these two shows a small deviation—this means the simulated model can reflect the real conditions of the selected research site (Figure 5).

The meteorological data, including air temperature and relative humidity (RH), are recorded every minute, and the information of the used instrument is shown in Table 3.

### 2.3. Numerical Simulation by ENVI-met

As technology has developed, numerical simulation has been widely used, mainly due to its capability of calculating meteorological conditions, vegetation and soil processes and building surface energy fluxes within the outdoor urban environment across a serious of urban configurations. To date, ENVI-met software has been the most accurate for assessing the outdoor thermal environment [19,20,21,22,23]. With this software, all modelling systems must be compared to collected field survey data to determine their ability in supplying accurate output data under the urban environment.

As opposed to other software, vegetation, including trees and grass, is grouped in accordance with its size, type and leaves, which are all essential factors for affecting radiation and reflection. As ENVI-met analyzes vegetation based on leaf area density (LAD) and not leaf area index (LAI), the following equation is used to show the relationship between the two parameters:

(1) $$\mathrm{LAI}=\overset{\;h}{\underset{0}{\int }}\mathrm{LAD}.z\;$$

where h is the height of the tree (m) and z is vertical grid size.

**Figure 6:**
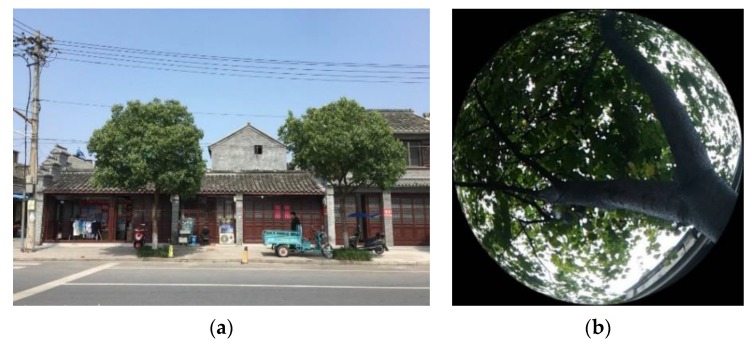
(**a**) A typical image of a camphor tree (**b**) Fisheye image of a camphor tree [18].

**Figure 7:**
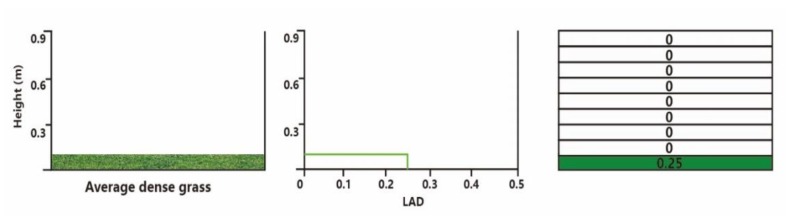
Schematic of grass database model for ENVI-met.

According to field measurements, in this study, the local border tree was the camphor tree (Figure 6). The detailed data of the tree was added to the ENVI-met plant database to fulfil this research (Table 4) [24]. The green grass used in this block is shown in Figure 7. The green grass used in this block is shown in Figure 7, in which the height of the grass is 0.25 m and the LAD is 0.25 m^2^/m^3^.

**Figure 8:**
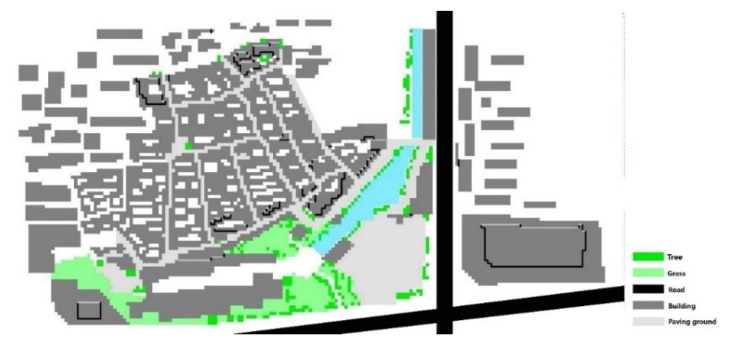
The simulated model in ENVI-met.

The initial input data of the various meteorological elements used in the ENVI-met simulation are shown in Table 5. In this study, the whole simulation was conducted over a 48-h period, starting from midnight 00:00 on 30 July 2016, with calculations every 1 min. The simulation results were output on an hourly basis. The simulated model in ENVI-met is shown in Figure 8.

### 2.4. The Validation Between Measured and Simulated Data

To quantify the gap between the measured and simulated data, the root mean square error (RMSE) was calculated for each selected point. The index RMSE is a significant factor for calculating the error and has been widely used in previous studies [24,25]. If the RMSE can reach or approach zero, the most accurate model can be achieved. A lower RMSE value means that the simulated results are within the measured value. Figure 8 shows the RMSE values between the measured and simulated air temperature and relative humidity.

**Figure 9:**
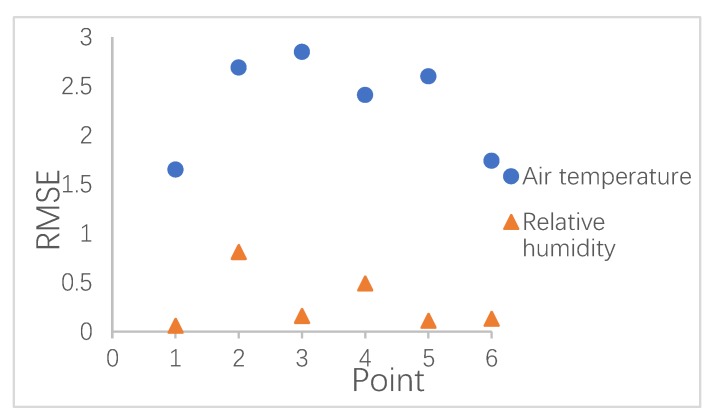
The root mean square error (RMSE) between measured and simulated data.

**Figure 10:**
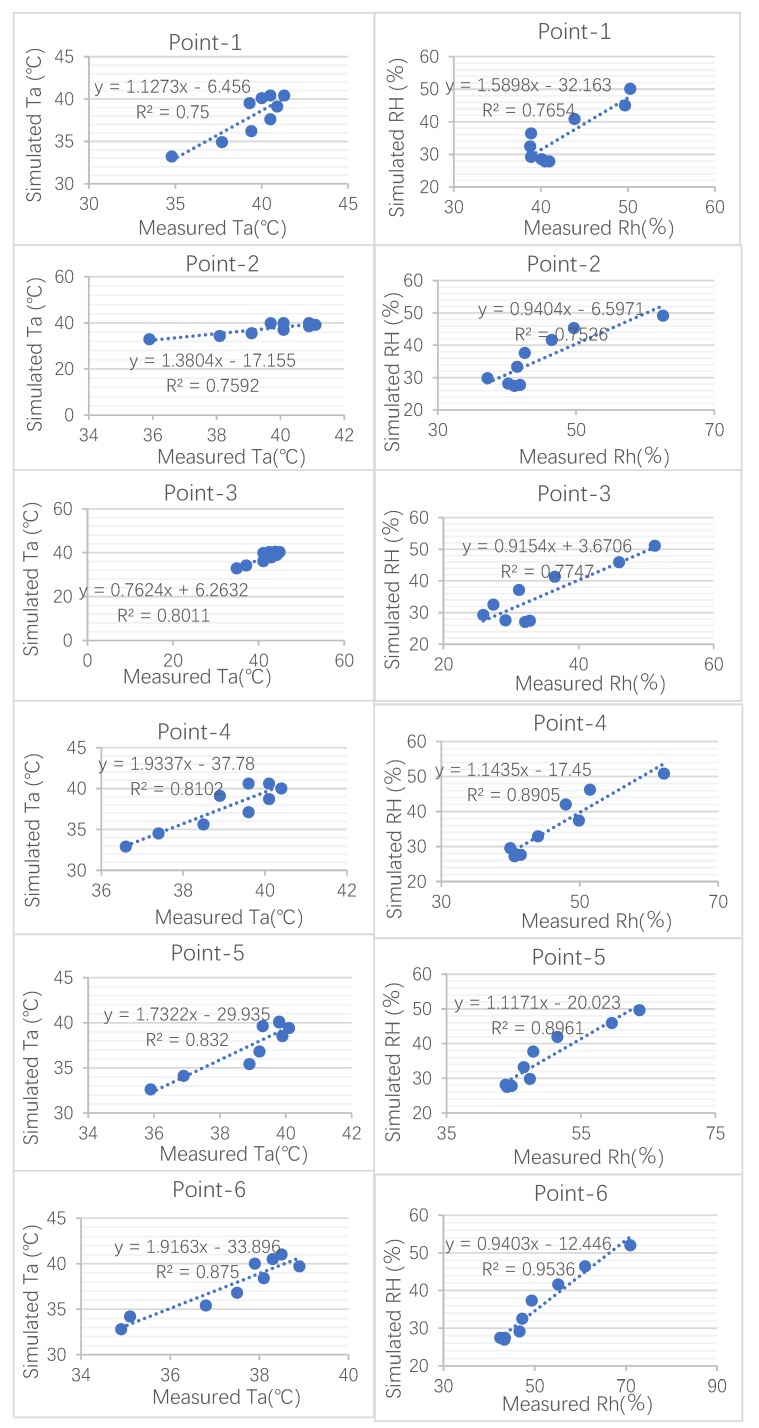
The correlation between the simulated data and measured data on 30 July (Ta air temperature, RH relative humidity) [18].

**Figure 11:**
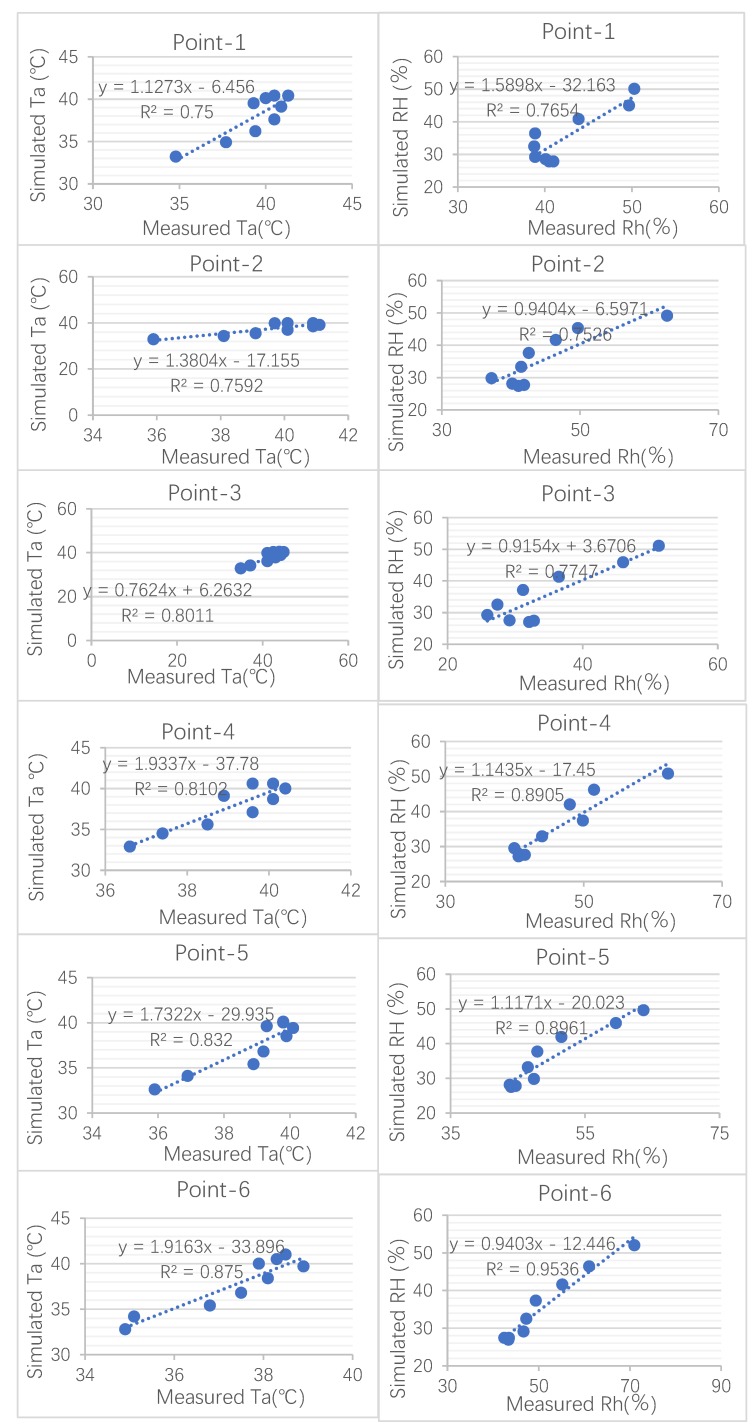
The correlation between the simulated data and measured data on 31 July (Ta air temperature, RH relative humidity) [18].

As is shown in Figure 9, point-3 had the highest error in the daytime, which reached 2.85 °C. This error can be attributed to the position of the recording machine. Due to consideration for tourist safety, the data instrument was not fixed in the middle of the street but instead was fixed along the sidewalk. In addition, the accuracy of the relative humidity was better than that of air temperature. As well as the index RMSE, analyzing the correlation between the simulated and measured data was another step in evaluating the numerical simulation. To test the validity of the simulated model, the measured data were fitted with the simulated data by linear regression. A good liner regression was obtained, as shown in Figure 10 and Figure 11, where R^2^ values for air temperature of this region ranged from 0.75 to 0.9578, while those for relative humidity were between 0.7518 and 0.9813. In reality, the deviation between simulated and measured data may have been caused by anthropogenic heat from human activity. These results are similar to or even smaller than those from previous studies [26,27,28]. The final linear regression values proved that ENVI-met is valuable software that can be used to fulfil future research as part of this study.

### 2.5. The Thermal Index for Assessing Humans’ Thermal Sensation

Current research in both climatology and biometeorology has made a full contribution to developing human thermal comfort indices, such as standard effective temperature (SET) [29], predicted mean vote (PMV) [30], physiologically equivalent temperature (PET) [31] and so on. Based on human energy balance, the PET index has been widely used in outdoor spaces and is defined as a typical indoor setting for the heat budget of the human body balanced with the same core and skin temperatures as those in outdoor space; moreover, it uses the very simple unit (℃) as the thermal indicator of the outdoor microclimate. A study has shown the thermal sensation classification for humans in the hot-summer and cold-winter climate zone by collecting data and questionnaire responses, as is shown in Table 6 [18]. In our study, we used it for assessing the thermal conditions of our research site.

## 3. Results

**Figure 12:**
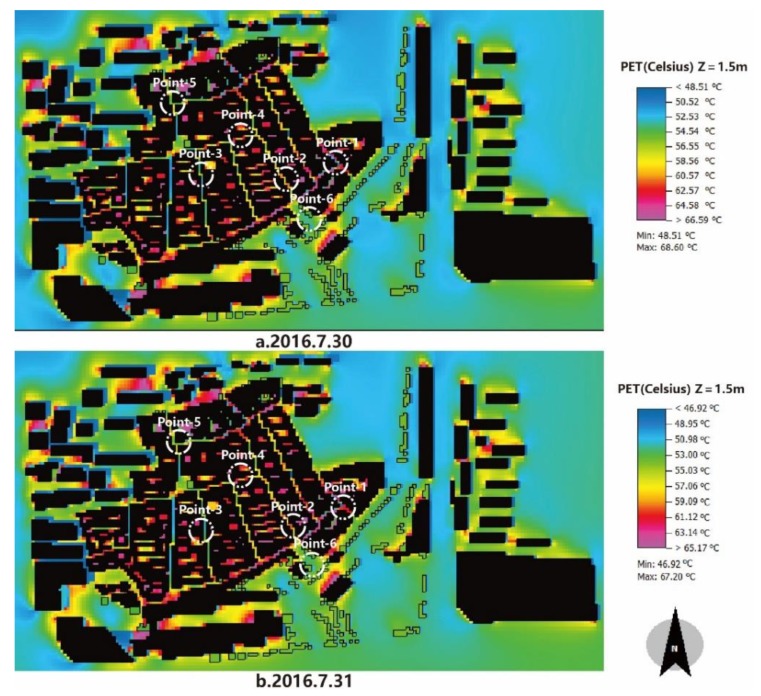
Spatial distribution of PET at 3:00 pm during the two measured days. (**a**): First day; (**b**): Second day.

Considering the microclimate in the selected points under the base case, the hottest time occurred at 3:00 pm. As can be seen in Figure 12, the PET values in the two measured days at 3:00 pm were very high.

Figure 12a indicates the thermal sensation in the first measured day, in which the lowest PET was 48.51 °C and the highest value reached 68.60 °C. According to the thermal sensation for hot-summer and cold-winter climate zones of southern China, nearly the entire block was within the “hot” and “very hot” zones. Due to the different weather conditions in the two measured days, we used the published data from the local weather station. It was clear that the average air temperature in the first measured day was 1 °C higher than that in the second day, which directly led to a higher PET value in the daytime. Figure 12b shows that the PET values at 3:00 pm ranged from 46.92 °C to 67.20 °C. It was even lower than the first day; however, the entire region was still in the “hot” and “very hot” zones. According to previous studies, it is evident that a stronger cooling effect is obtained with a higher background daytime air temperature [32,33]. In addition, the effect of paving material with higher albedo is better at reducing surface temperatures on sunny days than on cloudy days [34]. It has also been shown that the positive effect of vegetation on hot, sunny days is two times higher than on cold, cloudy days [35]. In this study, the hottest time appeared at 3:00 pm; therefore, the PET at 3:00 pm on 30 July was selected for calculations for further study.

**Figure 13:**
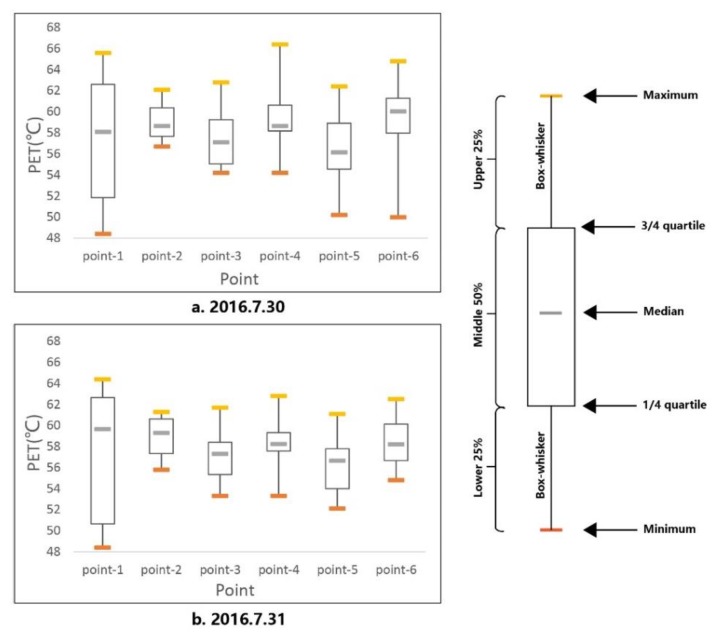
The PET of the selected points at 3:00 pm. (**a**): First day; (**b**): Second day.

The box plot figure, reflecting the comparison among a series of group data, is established under the existing scenario (Figure 13), in which the extreme summer PET is within 48.4 to 67.2 °C in all selected points.

## 4. Discussion

### 4.1. Outdoor Thermal Environment Under New Cases

The simulation in this study aimed to provide a comprehensive system for choosing the most effective strategy to improve outdoor thermal comfort in a commercial pedestrianized zone. For each parameter, hourly PET was carried out using the ENVI-met tool. The future strategies were modelled in four different cases. As explained above, the new models were simulated under the same microclimate conditions with the existing scenario. To be mentioned, the new cases are based on the local design specification [34], in which the buildings in a commercial pedestrianized zone will not be designed to exceed three stories, and the coverage ratio of vegetation will not be less than 25%. The first case (case-1) aims at understanding the detailed effect of increasing the building height. In the second case, the number of trees is increased to provide additional canopy coverage for this region. In the third case, the grass coverage ratio is provided to alleviate heat stress, also, the coverage ratio is same as that in case 2. The last case aims at researching the cooling effect of the paving material with a higher albedo. Table 7 shows the cases, modelling the future scenarios.

**Figure 14:**
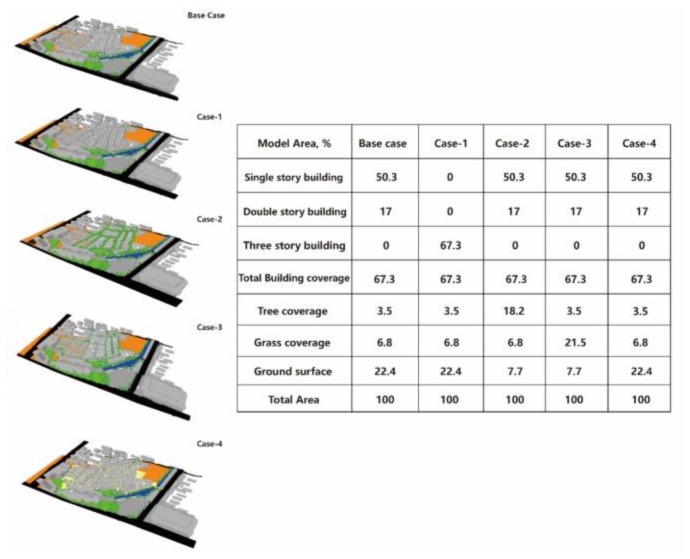
Various types of parameters in new configurations.

Based on the current conditions of the simulated model, the existing case was defined as the base, and four new cases were developed to compare and evaluate the cooling effects of different parameters (Figure 14).

The designed scenarios included the following: The first scenario (the base-case) was the base condition, which was derived from current conditions with building and vegetation coverage ratios of 67.3% and 10.3%, respectively. In this case, there were no three-story buildings in this region. Single-story buildings covered 50.3% and double-storied buildings occupied 17%. In addition, tree coverage was 3.5% and grass coverage was 6.8%, which was much less than the local design specification (25% of vegetation). The second scenario (case-2) aimed at increasing building height to understand the cooling effect—the three-story building coverage ratio was increased to 67.3% of this area. In the third scenario (case-2), the tree coverage ratio was increased to 18.2%. The fourth scenario (case-3) was applied by increasing the grass coverage ratio to improve human thermal sensation of comfort and to evaluate the cooling effect. The last scenario (case-4) was conducted by changing the existing paving material to material with a high albedo to understand the cooling effect.

**Figure 15:**
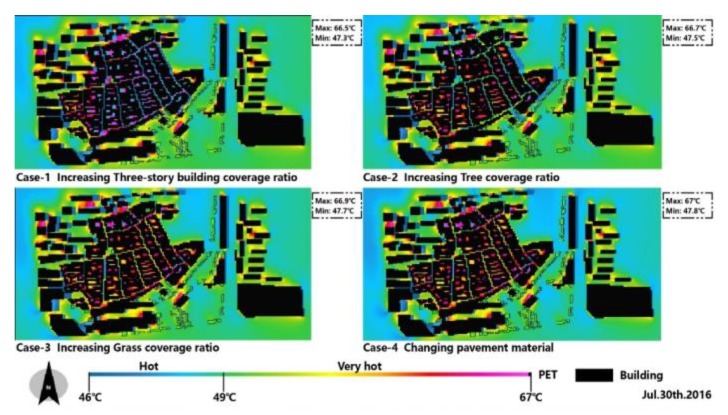
New PET distribution in different cases at 3:00 pm in the first measured day.

Under the new cases, the PET improvement appeared in the whole region. The peak time at daytime (3:00 pm) during the measured period is also compared at the pedestrian level (a height of 1.5 m) (Figure 15).

**Figure 16:**
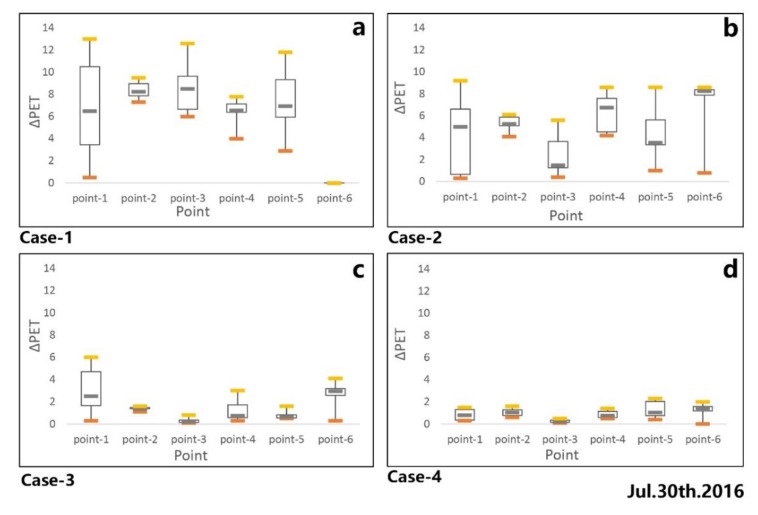
Cooling effect under the new cases at 3:00 pm. (**a**): case-1; (**b**):case-2; (**c**): case-3; (**d**): case-4.

The corresponding value and impact of different parameters is estimated as:

(2) ΔPET =PET−PETs 

where PET represents humans’ thermal comfort in this region and PET_S_ is the new thermal comfort after changing different parameters. These comparisons are conducted to evaluate and understand the cooling effect of different parameters, as is shown in Figure 16.

These PET results were processed with the same range and conditions to obtain a fair comparison between the base case and the new cases. On the first measured day (30 July 2016), increasing building height not only supplied more shading for humans but also impeded solar radiation during the daytime. The impact of shading on thermal comfort was quantified by calculating the difference of PET between canyon space and open space (Figure 16a), in which a positive ∆PET (thermal comfort improvement) from 0.8 to 12.6 °C was achieved. Meanwhile, an invalid effect was found in the open space during the daytime. The cooling function of trees is through transpiration and by providing shading to prevent solar radiation, thus improving thermal comfort during the daytime. It is clear that the reduction of PET was achieved both in canyon space and open space (Figure 16b), in which the ∆PET values ranged from 0.3 to 9.2 °C. Figure 16c shows the effectiveness of the grass, the cooling effect of which, unlike trees, depends only on transpiration, which leads to a worse cooling result compared with trees. Compared to other cases, as is shown in Figure 16d, changing the pavement material with high albedo material also improved thermal comfort, but the extent was limited.

In our study, numerical simulations were conducted to evaluate the correlation between different parameters and human thermal sensation of comfort in hot summers. The current results show the spatial distribution of human thermal comfort modification through the synergistic effect of different parameters in general. The next section provides a more thorough description about the effect of each parameter.

### 4.2. Correlation Between Different Parameters and Humans’ Thermal Comfort

**Figure 17:**
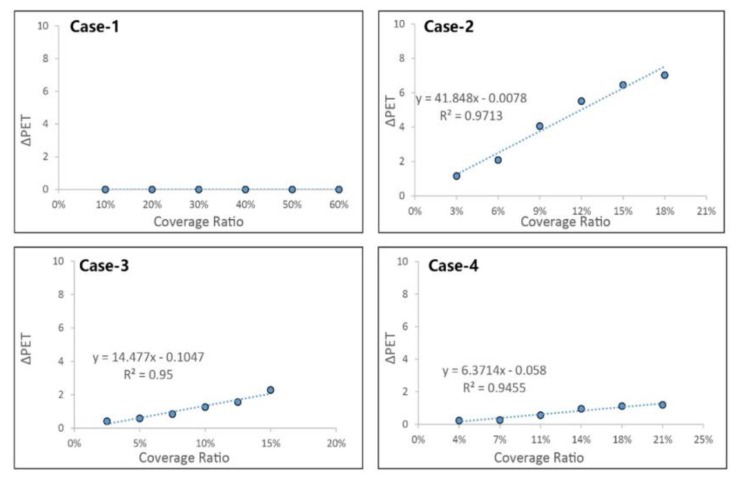
Correlation between thermal comfort and coverage ratio of different parameter in open space at 3:00pm.

As mentioned above, the whole area consisted of open space and canyon space. The effectiveness of different parameters in open space is shown in Figure 17.

Different parameters had different effects in improving thermal comfort at the hottest times during the measured period. Multiple regression analyses of ∆PET values of the different parameters at 3:00 pm were conducted to assess the contribution of these to improving thermal comfort. The correlation coefficient (R^2^) between PET and different parameters served to describe the proportion that could be explained by the variables of the regression model [35]. A strong positive correlation was found between the percentage of trees (case-2) and ∆PET, with the correlation coefficient being 0.9713. It was observed that a 3% increase in the coverage ratio of trees reduced PET by 0.78 °C at the hottest time during the extreme summer. Meanwhile, an irrelevant relationship between building height and ∆PET was observed (case-1), which meant that increasing building height could not effectively improve thermal comfort in open spaces. The other parameters, including grass (case-3) and pavement material (case-4), could reduce PET, but the extent was limited.

**Figure 18:**
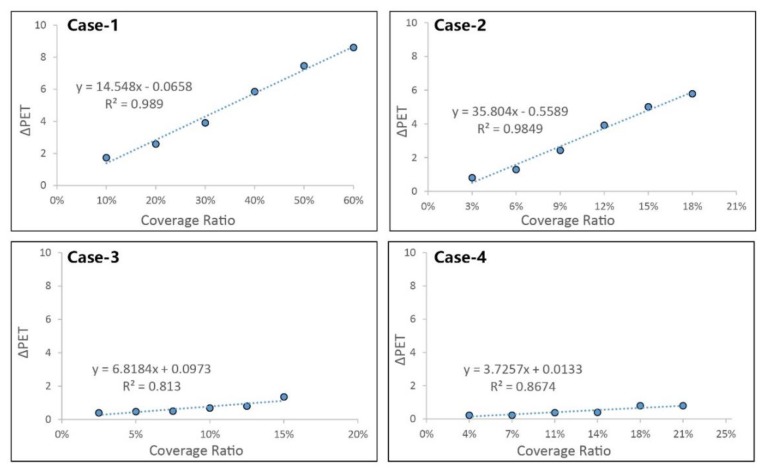
Correlation between thermal comfort and coverage ratio of different parameter in canyon space at 3:00 pm.

Figure 18 shows the changing situation in canyon space, where, unlike in open space, the heat stress could be alleviated in all the new cases. Increasing building height could effectively reduce PET (case-1) during the daytime. Based on its values, it could be seen that a 10% increase in the coverage ratio of the three-story building could decrease the PET by 1.3 °C. This effect could be attributed to the shading in the street. In addition, increasing the tree coverage ratio could also lead to thermal comfort improvement (case-2). An increase of 3% in the coverage ratio of trees could result in a decrease of 0.9 °C in PET values. The effect of grass (case-3) occurred through the reduction of reflected radiation to improve thermal comfort, but the result was poor, as was the case with increasing the coverage ratio of grass. Changing the pavement material to high albedo material (case-4) could decrease diffuse reflection, which could improve thermal comfort, but the simulated result was not obvious.

**Figure 19:**
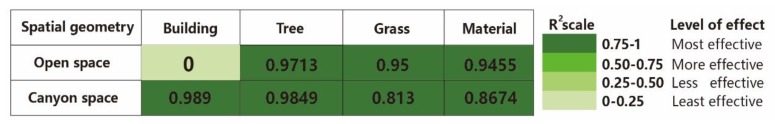
Correlation between different parameters and thermal comfort.

Based on the multiple regression results, designers and policy makers can choose the best way to redesign this block and improve the outdoor energy efficiency. The final results, which indicated the correlation between each parameter and human thermal comfort, can be classified as different types of choices (Figure 19).

In terms of thermal comfort, the PET index in this study was used to calculate the energy balance of the human body, which is directly affected by surrounding factors. The numerical simulation results suggest to us that a better prediction of the effects of pedestrian block renewal can improve humans’ thermal comfort by choosing the best strategy, thus helping to reduce outdoor energy consumption and improve outdoor thermal health for humans.

## 5. Summary and Conclusions

This study aimed at investigating the cooling effects of different parameters (building height, tree, grass and pavement material) on reducing heat stress during hot summers. In order to understand it well, field measurements and numerical simulations were conducted to evaluate human thermal comfort in this region, which can help designers and policy makers have a deeper understanding of the correlation between thermal comfort and different parameters and thus choose the best strategy to improve thermal comfort and the outdoor thermal environment. While some conclusions are common sense, quantitative results are still necessary, especially in the commercial pedestrian block. In this study, the distributions of PET values at 3:00 pm showed that the thermal environment in the block can be improved with new design parameters, where ∆PET ranges had maximum and minimum values at 12.6 and 0.3 °C, respectively. According to the final simulated results, regression analyses indicated that the most effective strategy in improving thermal comfort in the open space is to increase the coverage ratio of trees. In the canyon space, the most effective strategy is to increase the coverage ratio of three-story buildings.

The final outcome of this study can provide a comprehensive standard for designers and policy makers. Only by increasing the integration of municipal actors and researchers can mitigation actions be developed to improve the livability and quality of the commercial pedestrianized zone as well as the human thermal sensation of comfort. The following is suggested:

1. Increasing average building height and three-story building coverage ratio in canyon space can largely improve people’s thermal sensation.
2. Increasing the tree coverage ratio in open space can largely reduce heat stress at daytime. Our research further shows there is a strong correlation between the reduction of PET and increases in the tree coverage ratio.
3. Reducing the percentage of hardened ground in the commercial zone would be beneficial. In this site, local managers can use lawn or grass to replace the existing ground surface.

In addition, the limitations of this study cannot be neglected. Firstly, even though the ENVI-met software utilized has very high accuracy in forecasting the outdoor thermal environment, the deviation between measured and simulated data still cannot be ignored. Additionally, this work only simulates a single kind of tree, which in the real world may not be present. Thus, in future studies, we should consider different kinds of trees. Also, in future studies, we will overcome the mentioned limitations and provide a briefer way to improve humans’ thermal health in the hot summer.

## Figures and Tables

**Table 1:** Meteorological data on 30 and 31 July 2016.

Time	Weather	Maximum Air Temperature (°C)	Minimum Air Temperature (°C)	Wind Velocity (m/s)	Wind Direction
30 July	Sunny	37	27	2.0	South–East
31 July	Cloudy	36	27	2.9	South–East

**Table 2:** Characteristics of the selected points [18].

Point	Site Characteristic	Surface Type	Shade	Aspect Ratio (H/W)
1	North-West oriented street	Grey brick	Yes	4.6
2	North-West oriented street	Grey brick	Yes	1
3	North-South oriented street	Grey brick	Yes	2.3
4	East-West oriented street	Grey brick	Yes	2.3
5	North-South oriented street	Grey brick	Yes	2.75
6	Open space	Grey granite	No	0.33

**Table 3:** Introduction of the measured instruments.

Instrument	Mode	Accuracy	Range	Interval	Sensor
Relative Humidity (RH)	Automatic	±5% RH	10%–95% RH	60 s	TR-70wf
Air Temperature	Automatic	±0.5 °C	0–+55 °C	60 s	TR-70wf

**Table 4:** The leaf area density (LAD) distribution of the tree [18].

Height(m)	1	2	3	4	5	6	7
LAD	0	0	0	2.0	2.95	2.95	2.0

**Table 5:** Initial simulated data for ENVI-met.

Input for Configuration File	Value
Start simulation	0:00, 30 July 2016
Total simulation time	48 h
Wind speed in 10m (m/s)	2.0
Wind direction	145
Initial air temperature (°C)	37
Relative humidity (%)	45
Roughness length	0.1
Number of x grids	200
Number of y grids	100
Number of z grids	20
Size of the grid in dx (m)	3
Size of the grid in dy (m)	3
Size of the grid in dz (m)	2
Albedo ground	0.4
Albedo roof	0.2
Albedo wall	0.3

**Table 6:** The distribution of humans’ thermal sensation in hot-summer and cold-winter area [18]. PET = physiologically equivalent temperature.

Thermal Sensation	PET(°C)
Very Cold	<−4
Cold	−4–3
Cool	3–11
Slightly Cool	11–19
Neutral	19~26
Slightly Warm	26~34
Warm	34~42
Hot	42~49
Very Hot	>49

**Table 7:** The tested scenarios with new strategies.

Scenario	Selection Strategies
Case-1	Increasing average building height.
Case-2	The trees are implanted in the research site.
Case-3	The grass is implanted in the research site.
Case-4	Changing the paving material with a high albedo.
